# Ketogenic diet treatment in diffuse intrinsic pontine glioma in children: Retrospective analysis of feasibility, safety, and survival data

**DOI:** 10.1002/cnr2.1383

**Published:** 2021-05-03

**Authors:** Alexandre Perez, Elles van der Louw, Janak Nathan, Moatasem El‐Ayadi, Hadrien Golay, Christian Korff, Marc Ansari, Coriene Catsman‐Berrevoets, Andre O. von Bueren

**Affiliations:** ^1^ Cansearch Research Platform for Pediatric Oncology and Hematology, Faculty of Medicine, Department of Pediatrics, Gynecology and Obstetrics University of Geneva Geneva Switzerland; ^2^ Division of Pediatric Oncology and Hematology, Department of Women, Child and Adolescent University Hospital of Geneva Geneva Switzerland; ^3^ Department of Dietetics, Erasmus MC Sophia Children's Hospital University Medical Centre Rotterdam The Netherlands; ^4^ Department of Neurology Shushrusha Hospital Mumbai India; ^5^ Department of Pediatric Oncology, National Cancer Institute Cairo University Cairo Egypt; ^6^ Department of Pediatrics, Obstetrics and Gynecology, Pediatric Neurology Unit University Hospital of Geneva Geneva Switzerland; ^7^ Department of Pediatric Neurology Erasmus MC Sophia Children's Hospital, University Medical Centre Rotterdam The Netherlands

**Keywords:** CNS tumors, gliomas, brainstem, neuro‐oncology, nutrition, pediatric hematology/oncology, tumors, brain

## Abstract

**Background:**

Diffuse intrinsic pontine glioma (DIPG) is one of the most devastating diseases among children with cancer, thus novel strategies are urgently needed.

**Aims:**

We retrospectively evaluated DIPG patients exposed to the carbohydrate restricted ketogenic diet (KD) with regard of feasibility, safety, and overall survival (OS).

**Methods and results:**

Searches of MEDLINE and Embase identified five hits meeting the search criteria (diagnosis of DIPG and exposure to KD). One additional case was identified by contact with experts. Individual patient data were extracted from publications or obtained from investigators. The inclusion criteria for analysis of the data were defined as DIPG patients who were exposed to the KD for ≥3 months. Feasibility, as described in the literature, was the number of patients able to follow the KD for 3 months out of all DIPG patients identified. OS was estimated by the Kaplan‐Meier method.

Five DIPG patients (males, n = 3; median age 4.4 years; range, 2.5‐15 years) meeting the inclusion criteria were identified. Analysis of the available data suggested that the KD is generally relatively well tolerated. Only mild gastro‐intestinal complaints, one borderline hypoglycemia (2.4 mmol/L) and one hyperketosis (max 7.2 mmol/L) were observed. Five out of six DIPG patients identified adhered for ≥3 months (median KD duration, 6.5 months; range, 0.25‐2 years) to the diet. The median OS was 18.7 months.

**Conclusion:**

Our study provides evidence that it may be feasible for pediatric DIPG patients to adhere for at least 3 months to KD. In particular cases, diet modifications were done. The clinical outcome and OS appear not to be impacted in a negative way. KD might be proposed as adjuvant therapy when large prospective studies have shown feasibility and safety. Future studies might ideally assess the impact of KD on clinical outcome, quality of life, and efficacy.

**Abbreviations**
DIPG: diffuse intrinsic pontine glioma
Dx: diagnosis
KD: ketogenic diet
MRI: magnetic resonance imaging
OS: overall survival
RT: radiation therapy

## 1 INTRODUCTION

Diffuse intrinsic pontine glioma (DIPG) is the most frequent brainstem tumor in the pediatric populations, counting for up to 80% of all pediatric brainstem tumors.^1^ DIPG is known to have a dismal prognosis with a median survival of approximately 11 months using the current standard of care.^2^

The standard treatment consists of involved‐field radiation therapy (RT); this approach frequently improves the symptoms and prolongs the patient's life by a few months. Using other treatment strategies in addition to RT—in particular chemotherapy—has not changed the poor outcome of DIPG patients.^1^

Given this devastating outcome, physicians treating children with DIPG are frequently confronted by the strong wish of the parents to complement the standard treatment of their children with additional strategies including alternative and complementary therapies. The use of alternative and complementary therapy in pediatric oncology patients appears to be common (in approximately 42% of patients) as reported before.^3^ Herbal teas, plant extracts, therapeutic vitamins, and diet seem to be the most commonly used alternative therapies in pediatric oncology patients.^3^ These treatment experiences, partly collected within clinical trials, are infrequently reported in the literature.

Based on the fact that tumors need significant amounts of glucose to sustain themselves, the hypothesis was postulated that undergoing a ketogenic diet (KD), a diet rich in lipids and low on carbohydrates, could help weaken the tumor and make it more susceptible to radiation therapy.^4^, ^5^, ^6^

When given a diet that is very restricted in carbohydrates, the body reacts by creating ketone bodies from fat in the liver. The ketone bodies are then distributed throughout the body and used by the cells as a source of energy. Interestingly though, glioblastoma cells appear to be incapable, unlike normal brain cells, of using ketone bodies as their primary source of energy and therefore weaken if the blood glucose levels decrease.^6^, ^7^

In vivo studies using mice support this hypothesis. The application of KD resulted in a reduction in the size of the tumor, and when used concomitantly with radiotherapy, the effect was even stronger.^4^, ^8^

The potential effect of KD in the management of DIPG patients seems to be of interest, as long as it does not provoke any significant adverse effects and is generally well tolerated by the patients without affecting their quality of life.

The KD has been traditionally used, as early as the beginning of the 20th century, to treat drug‐resistant epilepsy primarily in children, and has proven to be beneficial in reducing seizures.^9^, ^10^, ^11^ More recent studies have identified a potential use as a metabolic therapy against cancer.^4^, ^12^

The main goal of this retrospective study is to investigate the feasibility and potential adverse effects of KD used in patients with DIPG. The overall survival (OS) was explored in a descriptive manner, and the impact of KD on symptomatic improvement was qualitatively estimated.

## 2 PATIENTS AND METHODS

### 2.1 Search strategy, data collection, and extraction

We initially searched the literature to identify a maximum number of DIPG patients (without age restriction) exposed to KD. The systematized search was conducted on 16th of July 2019 using predefined search terms on PubMed and Embase. We used no language or date restriction. The search terms (for details please refer to supplementary file 1) were the following: (“Glioma”[Mesh] OR Glioma*[Tiab] OR “Brain Stem Neoplasms”[Mesh] OR Brain‐Stem‐Neoplasm*[Tiab] OR “Brain Neoplasms”[Mesh] OR Brain‐Neoplasm*[Tiab] OR brain‐cancer*[Tiab] OR Diffuse Intrinsic Pontine Glioma*[Tiab] OR DIPG[Tiab]) AND (“Diet, Carbohydrate‐Restricted”[Mesh] OR low carbohydrate diet*[Tiab] OR Ketogen*[Tiab] OR keto‐diet*[Tiab] OR ketotic*[Tiab] OR carbohydrate restricted diet*[Tiab]).

**FIGURE 1:**
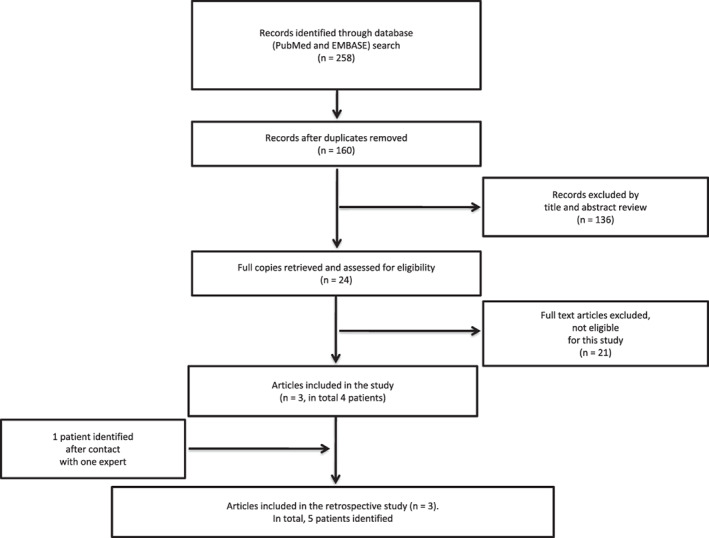
PRISMA diagram: Summary of the literature search from initial results to final number of patients identified. Our search identified 258 publications, which was reduced to 160 with the removal of duplicates. Then 136 articles out of 160 were excluded by title and abstract review (diagnosis other than DIPG in most cases). Out of the 24 articles remaining, 21 were excluded after full text review (articles not reporting on DIPG patients). The three publications included in the retrospective study consisted of one prospective study,^16^ one letter to the editor,^18^ and one conference abstract.^17^ The prospective study included three patients of which only two were exposed ≥3 months to the ketogenic diet (case number = 1 and 2). The conference abstract included one patient (case number = 3) and contact with one expert allowed the addition of another patient (case number = 4). The letter to the editor included information about one patient (case number = 5)^18^

The step‐by‐step process of the search is shown in Figure 1, inspired by the PRISMA (preferred reporting items for systematic reviews and meta‐analyses) statement.^13^ We took the following steps to make our search systematic: the search was conducted in the most comprehensive way using broad search terms in order to optimize the spectrum of the search. It was performed by two independent reviewers (AP and HG), who agreed on the inclusion/exclusion criteria of the search in a dedicated protocol prior to the search (search criteria were patient diagnosed with a DIPG and exposed to KD).

On the other hand, no quality assessment of the results was performed, and no protocol was published prior to conducting the search. It is therefore not considered as a systematic review.

Once the studies of interest were identified, we contacted the authors to get the updated anonymous data about their cases. All the patients who met the inclusion criteria were included in the study. Individual patient data were extracted from publications or obtained from investigators.

### 2.2 Eligibility criteria

The eligibility criteria for this retrospective study were defined as patients diagnosed with DIPG exposed to KD for ≥3 months. The eligibility criteria including the exposition to KD for ≥3 months were chosen before starting the study also for sake of comparability as clinical outcome/response evaluation upon exposition to KD is usually performed after a KD treatment of 3 months.^14^

### 2.3 Feasibility assessment

Feasibility was defined—comparable with previous studies^15^, ^16^—as the number of DIPG patients who were able to use the KD for ≥3 months. The feasibility assessment is based on the comparison of DIPG patients exposed to a KD for ≥3 months with all identified DIPG patients exposed to KD.^16^

### 2.4 Safety assessment

Safety of the KD was evaluated and the main complications and adverse events potentially related to the diet were assessed. All adverse events were defined and graded according to the common terminology criteria of adverse events (CTCAE) version 4.0.

### 2.5 Assessment of potential clinical benefit

Clinical improvement in relation with the KD was assessed qualitatively only, more specifically the clinical improvement was not investigated using standardized procedures and assessments. In this retrospective study, the symptomatic improvement was estimated by the treating physician/team of the patient.

### 2.6 Statistics

The OS was estimated by the Kaplan‐Meier method. This analysis as well as additional descriptive statistics was done using IBM SPSS Statistics for Windows, version 26 (IBM Corp., Armonk, New York).

## 3 RESULTS

The process of evaluation of the articles is illustrated in Figure 1. We identified 258 publications using the search terms. After removal of duplicates, the number of hits identified was reduced to 160. Title and abstract review resulted in the exclusion of 136 publications. Full review of the remaining articles resulted in three publications fulfilling the inclusion criteria. After detailed evaluation of the three publications, five patients were identified, four of the five identified cases met the inclusion criteria, and one additional patient, not published thus far, was added after contact with one expert; thus resulting in five cases fulfilling the inclusion criteria.

### 3.1 Patient characteristics

Among the five patients, three were males, with a median age of 4.4 years (range, 2.5‐15 years). Patients 1 and 2 were published before,^16^ patients 3 and 4 were treated at the same institution (one patient reported in a meeting abstract),^17^ and patient 5 was reported elsewhere.^18^ All diagnoses were confirmed by magnetic resonance imaging (MRI) and no biopsies were performed.

The time between the diagnosis and the start of the KD differed (Table 1) as strategies included a start of the diet directly after diagnosis (case 5) vs initiating the diet after disease relapse/progression (case 1, 2, 3, and 4), as described elsewhere for patients 1 and 2.^16^

**TABLE 1:** DIPG patients characteristics and treatment modality

No.	1	2	3	4	5
PUBMED ID	30 484 948	30 484 948	Conference abstract	Unpublished	30 767 367
Age at diagnosis (years)	4.4	14.4	15.0	3.7	2.5
Gender	M	M	F	F	M
Diagnosis	DIPG (MRI)	DIPG (MRI)	DIPG (MRI)	DIPG (MRI)	DIPG (MRI)
Type of KD used (for details please refer to supplementary file 2)	KDT/MCT	KDT /MCT	Classical KD	Classical KD	Modified Atkins diet
Time between Dx and start of KD (months)	11	12.2	3	6	Shortly after initial diagnosis
Reason for KD	KD‐study participation	KD‐study participation	Complementary treatment	Complementary treatment	Complementary treatment
Duration of KD (months)	3	6.5	6	16	24
Primary treatment	TMZ (tablets)—RT	TMZ (tablets)—RT Prednisolone	Observation only	RT	HIT‐SKK chemotherapy Proton therapy
Treatment at progression / relapse	KDT /MCT only	KDT /MCT, chemotherapy, re‐irradiation	Classical KD only	Classical KD only	Modified Atkins diet was stopped Second RT
Overall survival rate (months)	16.5	18.7	9	22	30

Abbreviations: Dx, diagnosis; F, female; HIT‐SKK chemotherapy, chemotherapy often administered to young children [SKK, Säuglinge und Kleinkinder] with brain tumors [HIT, Hirntumor] in Germany, please refer to the original description of the case 5;^18^ KD, ketogenic diet; KDT, ketogenic diet therapy; M, male; MCT, medium chain triglyceride; MRI, magnetic resonance imaging; RT, radiotherapy; TMZ, temozolomide.

The patients were treated using different types of KD. The details about the diet of each patient are available in supplementary data/file 2.

Except for patient 3 who was only treated with the KD, all other patients were concomitantly or before/after the KD treated with standard treatment (radiotherapy and chemotherapy). Timeline as well as the complete treatment details for each patient are shown in Table 1.

### 3.2 Feasibility

Our search of the literature and contact with experts allowed us to identify six patients with DIPG who were treated using KD. Out of those six patients, five were able to follow the KD for ≥3 months. All five were included in this study. The sixth patient experienced an unfavorable clinical course soon after the start of the KD and has died.^16^ Patients 1 and 2 with progressive DIPG were exposed for ≥3 months. Parents of patient 1 decided not to continue the KD longer than 3 months (duration of the feasibility study).^16^ Patient 2 continued the KD for 3.5 more months (total duration of 6.5 months). Patient 3 was on the diet for 6 months and patient 4 for 16 months. The KD was stopped approximately after 2 years in patient 5.^18^

### 3.3 Clinical outcome

We analyzed the estimation of the clinical improvement during the KD only as assessed by the treating physician/team (supplementary data/file 3). There were no data with regard to clinical improvement available for patients 1 and 2 as it was not an end point of the previous prospective study.^16^ In the case of patients 3 and 4, the clinical condition was assessed by the treating physician, and reported to be better under the diet, with substantial improvement of several symptoms. For patient 5, the treating physician estimated the clinical outcome to be unchanged when compared to the clinical situation before starting the diet (observation interval, 1 month).

### 3.4 Safety

Table 2 illustrates the adverse events (≥ grade 2) observed while the patients were on the diet.

**TABLE 2:** Reported adverse events during treatment and related to KD

	Patients					
Adverse Event	1	2	3	4	5	% of total sample
Vomiting	Yes Related to the KD liquid formula	‐	‐	‐	Yes Causality with KD possible	40
Food refusal	Yes Related to the liquid formula	‐	‐	‐	Yes Linked to the vomiting Causality with KD possible	40
Fatigue	Yes Causality with KD at least partly possible	Yes Causality with KD at least partly possible	‐	‐	Yes Causality with KD possible	60
Headache	‐	‐	‐	‐	‐	0
Constipation	‐	Yes Related to KD	‐	‐	Yes Causality with KD possible	40
Inability to swallow	‐	Yes Related to the tumor	‐	‐	‐	20
Hyperketosis	−7.2 mmol/L (day 3)−6.7 mmol/L (days 18, 27, 57) Related to KD −7.2 mmol/L (day 26)	‐	‐	‐	No The maximum concentration was 5.8 mmol/L	20
Hypoglycemia	2.4 mmol/L (day 3, in the morning) Related to KD	‐	‐	‐	No Minimum blood glucose level was 3.4 mmol/L Related to KD	20
Increased serum lipid levels	No	No	‐	‐	Triglycerides were high High total cholesterol: 12.40 mmol/L (n range: 2.91‐5.36) Probably related to KD	20
Increased serum uric acid	No	No	‐	‐	Yes A few elevations, max 307 μmol/L (normal range: 100‐282) Causality with KD possible	20
Acidosis	No	No	‐	‐	One metabolic acidosis of pH 7.29 (n: 7.38‐7.42) Linked to the diet but within the tolerated range according to treating neurologist	20
Renal stones	No	No	‐	‐	No But chronic nephrocalcinosis since start of the KD Probably related to the KD	0
Increased rate of infections	No	No	‐	‐	One port‐a‐cath infection. (staph. Epidermidis cellulitis) Causality with KD possible	20
Gallstone formation	No	No	‐	‐	No	0
Dehydration	No	No	‐	‐	No	0
Significant elevation of liver enzymes	No	No	‐	‐	ALAT at 22 unit/L once (normal range: 0–19 unit/L) Causality with KD possible	20
Pancreatitis	No	No	‐	‐	No	0
Weight loss/gain	No	No	‐	‐	No	0

Abbreviations: ALAT, alanine aminotransferase; KD, ketogenic diet.

Patient 1 experienced vomiting, fatigue, and food refusal. These symptoms were mainly due to the liquid diet formula and disappeared when the diet was changed to a solid KD. Patient 2 suffered from fatigue, constipation, and an inability to swallow. Accordingly, the KD was given by tube feeding. Ketone and glucose levels were within the target range for all patients except patient 1, who experienced high ketosis of 7.2 mmol/L in blood (hyperketosis defined as >6 mmol/L) three times and one borderline glucose level of 2.4 mmol/L (hypoglycemia defined as <2.5 mmol/L) while on the diet. Patients 3 and 4, whose clinical condition improved under the diet, did not experience any documented adverse events while undertaking the diet (no hospitalizations were necessary).

Patient 5, who underwent the diet for 2 years, experienced symptoms like vomiting, constipation, food refusal and fatigue. Blood analyses showed one measurement of high total cholesterol, a slight elevation of serum uric acid and one acidosis linked to the diet but within the tolerated range according to the treating neurologist. This patient also suffered from one port‐a‐cath infection and chronic nephrocalcinosis. Overall, no severe adverse effects were documented in any of the patients.

### 3.5 Explorative estimation of the OS

We assessed the OS in a clearly explorative manner. Of the five patients who underwent the diet for ≥3 months, their OS was relatively favorable (median OS, 18.7 months, range, 9 months‐30 months), as illustrated in Table 1.

## 4 DISCUSSION

In this study, we aimed to increase the knowledge about the use of KD in the management of the very lethal pediatric cancer DIPG by assessing the feasibility and safety of the diet as well as the potential impact of the diet on clinical benefit and OS of the patients. After the identification of six patients mainly through a literature search, with five of them meeting the inclusion criteria, we were able to evaluate these different parameters.

Our analysis provides evidence that the KD might be feasible, as five out of six patients were able to undertake the diet for at least 3 months. One patient reported in the previous publication^16^ was not able to stay on KD—which was started at disease progression—for 3 months as the patient experienced an unfavorable clinical course and has died rapidly.

Starting the KD after relapse/disease progression—as it was the case for all patients except for patient 5—is less promising as with the reduced life expectancy,^19^ the treatment duration becomes more limited. Besides, there is the ethical dilemma of assigning an active treatment/diet plan—with the required investigations and clinical visits—to patients who are followed‐up by palliative care with modest medical interventions and appointments.^20^ Most importantly, according to the current literature, the KD appears to be more promising when used in cancer patients with early stages of disease.^21^ However, based on our experience reported here, we are unable to conclude in an evidence‐based manner about the ideal timeframe to start a KD in DIPG.

Based on our limited data, we provide evidence that the KD used in DIPG might be safe as only minor adverse events were reported and no hospitalization was needed as a direct consequence of the diet. Four out of five children tolerated the KD without important side‐effects. One patient had borderline hypoglycemia and hyperketosis. Reported side effects like headache and fatigue, which were observed in some patients, were more likely due to the underlying disease itself rather than the diet. Systematic review from the literature supports the use of KD in children as the treatment by KD is a safe therapeutic option in children with refractory epilepsy.^22^

Our study also aimed to explore how the diet may affect the symptomatic improvement, overall providing no evidence for a negative impact on clinical outcome, and even transiently ameliorating it in some cases with improvements of some symptoms resulting in a better participation in the daily life activities.

Overall, some studies^23^, ^24^, ^25^, ^26^, ^27^ have shown that the KD is generally well tolerated by children and adults suffering from different types of brain tumors, using KD with different nutritional compositions and different onset of KD, hence supporting the results of our research.

Although outside of the principal aim of the study, the OS of the DIPG patients is reported here. The median OS of the five patients included in the study was 18.7 months, which is higher than a generally reported median OS of 11 months.^2^ Despite a potential bias and the small sample size, there is no evidence that KD negatively impacts the survival of patients with DIPG.

The OS was investigated in an explorative manner and the cohort included at least one long‐term survivor. The favorable outcome of this patient might be partly explicable by the favorable age of the patient (aged <3 years at diagnosis),^2^ as well by the fact that the patient was irradiated two times as this approach appears to be related to a better OS as reported before.^28^ Of note, the neuro‐radiologic diagnosis of DIPG was confirmed by central review for this patient. Due to the limited number of DIPG patients, missing information about molecular characteristics, and different treatments applied, no clear conclusion can be drawn with regard to the impact of KD on OS.

There are some limitations of this study that need to be discussed. First, the study was conducted in a retrospective manner, suggesting that some data could be missing or incomplete. Some adverse events are potentially underreported. Patients 3 and 4 were managed with a disease‐adapted follow‐up, aiming to minimize the medical appointments in order to maximize the time they can spend with their families and friends. Second, different types of KD were used depending on the patients and the treatment centers. Although they all fall under the definition of KD (high fat, low carbohydrate) and therefore induce similar metabolic changes, the KD formulas used differed as they had to be adapted to the patient's current state. This may imply more variability in the results. Several authors including us are using KD as an “umbrella term” for low carbohydrate, adequate protein, and high fat diets—corresponding to carbohydrate restrictive diets—which promote the utilization of fat for energy, in the form of ketones. Generally, due to the low adherence rate when using restrictive KD on cancer patients,^21^ less stringent KD (eg, modified Atkins diet) appears to be more reasonable when considering the general clinical situation of adult and pediatric cancer patients. In this context, testing for ketosis seems to be appropriate with the aim to monitor the KD and the patient compliance in order to see whether the patient needs more support or diet counseling.

Another limitation is, as mentioned earlier, the small sample size. Despite our efforts, we could only include five patients. One reason is that although most parents of DIPG patients seem initially very interested in the dietary treatment alternative, many of them may probably lose the interest when their children enter a palliative stage of the disease and starts irreversibly losing neurological functions.^16^ Another obvious reason is the poor survival, increasing the difficulties to investigate therapeutic options particularly at relapse or progression.^29^

A further limitation is the heterogeneity of the patient group with regard of the aims and objective of the use of KD. Patients 1 and 2 were included in a prospective trial investigating the feasibility and safety of the KD in recurrent DIPG patients.^16^ The treating physician was offering the KD to patients 3 and 4 with the intention to better control the disease. The family of patient 5 used the KD as alternative and complementary therapy aiming to treat the disease and to potentiate conventional treatments (radiotherapy and chemotherapy). In this situation, the treating physician and team offered a combined follow‐up led by the pediatric oncologist (oncologic disease) and pediatric neurologist (ketogenic diet) in order to ensure a complete follow‐up.

Lastly, the potential impact of KD on the clinical outcome was not assessed using standardized procedures but instead indirectly by the treating physician. Thus, we are unable to exclude a bias.

Our retrospective study provides evidence that it may be feasible for DIPG patients to adhere for ≥3 months to KD. In particular cases, diet modifications were necessary. The clinical outcome and OS appear not to be impacted in a negative manner. These results should encourage further studies on a larger scale; ideally assessing the KD in DIPG patients when started shortly after diagnosis in an observational prospective study. KD might be proposed as adjuvant therapy when large prospective studies have shown feasibility and safety. Future studies would ideally also investigate the effect of KD on clinical outcome, quality of life, and efficacy.

## CONFLICT OF INTEREST

The authors declare that the research was conducted in the absence of any commercial or financial relationships that could be construed as a potential conflict of interest.

## AUTHOR CONTRIBUTIONS

**Alexandre Perez:** Data curation; investigation; methodology; project administration; writing‐original draft. **Elles van der Louw:** Data curation; formal analysis; methodology; writing‐review and editing. **Janak Nathan:** Data curation; investigation; project administration; writing‐review and editing. **Moatasem Elayadi:** Data curation; formal analysis; methodology; writing‐review and editing. **Hadrien Golay:** Data curation; methodology; project administration; writing‐review and editing. **Christian Korff:** Conceptualization; formal analysis; methodology; writing‐review and editing. **Marc Ansari:** Project administration; resources; writing‐review and editing. **Coriene Catsman‐Berrevoets:** Conceptualization; data curation; writing‐review and editing. **Andre von Bueren:** Conceptualization; data curation; investigation; methodology; project administration; supervision; writing‐original draft.

## ETHICS STATEMENT

This study was considered as falling outside of the scope of the Swiss legislation regulating research on human subjects, so that the need for local ethics committee approval was waived (confirmed by the local ethics committee). Written informed consent was obtained from patients, parents, or legal guardians of all patients.

## Supporting information


**Appendix S1.** Supporting Information

## Data Availability

The data that support the findings of this study are available from the corresponding author upon reasonable request.
