# Supplementary material for: Ketogenic diet treatment in diffuse intrinsic pontine glioma in children: Retrospective analysis of feasibility, safety, and survival data
Source: Cancer Rep (Hoboken). 2021 May 3;4(5):e1383. doi: 10.1002/cnr2.1383 (PMC8551993; doi:10.1002/cnr2.1383)
Supplement: Supplementary file 1 — Appendix S1. Supporting Information [file CNR2-4-e1383-s001.zip › CNR2_1383_2021_03_08_Supplementary_data file 3.docx]

SUPPORTING INFORMATION

Clinical benefit estimation during the ketogenic diet treatment (supplementary data/file 3)

| No. | 1 | 2 | 3 | 4 | 5 |
| --- | --- | --- | --- | --- | --- |
| PUBMED ID | 30484948 | 30484948 | Conference abstract | Unpublished | 30767367 |
| Age at diagnosis (years) | 4.4 | 14.4 | 15.0 | 3.7 | 2.5 |
| Clinical benefit estimated by | Coping questionnaire | Coping questionnaire | Treating physician | Treating physician | Treating physician |
| Level of clinical improvement | / | / | Improved | Improved | Unchanged |
| Observations supporting the estimated clinical benefit | / | / | Patient improved her clinical condition. She managed to walk without support, write, read, dress. Speech improved  (could go back to school) | Patient improved her clinical condition. Asymptomatic (could go back to kindergarten) when under KD | No hospitalization due to the diet. Family was already using a low carbohydrate diet before diagnosis |

Abbreviation: KD: ketogenic diet
