# Supplementary material for: Ketogenic diet treatment in diffuse intrinsic pontine glioma in children: Retrospective analysis of feasibility, safety, and survival data
Source: Cancer Rep (Hoboken). 2021 May 3;4(5):e1383. doi: 10.1002/cnr2.1383 (PMC8551993; doi:10.1002/cnr2.1383)
Supplement: Supplementary file 1 — Appendix S1. Supporting Information [file CNR2-4-e1383-s001.zip › CNR2_1383_SUPPORTING_INFORMATION_supplementary datafile 1.docx]

SUPPORTING INFORMATION

Search information (supplementary data/file 1)

For Embase, the formula was as follow: ('glioma'/exp OR glioma:ab,ti,kw OR 'brain stem tumor'/exp OR "brain stem tumor*":ab,ti,kw OR "brain stem neoplasm*":ab,ti,kw OR "brain cancer*":ab,ti,kw OR "Pontine glioma*":ab,ti,kw OR DIPG:ab,ti,kw) AND ('low carbohydrate diet'/exp OR 'carbohydrate restricted diet*':ab,ti,kw OR 'low carb* diet*':ab,ti,kw OR 'ketogenic diet'/exp OR ketogen*:ab,ti,kw OR keto-diet*:ab,ti,kw) AND [embase]/lim NOT [medline]/lim
