# Supplementary material for: Ketogenic diet treatment in diffuse intrinsic pontine glioma in children: Retrospective analysis of feasibility, safety, and survival data
Source: Cancer Rep (Hoboken). 2021 May 3;4(5):e1383. doi: 10.1002/cnr2.1383 (PMC8551993; doi:10.1002/cnr2.1383)
Supplement: Supplementary file 1 — Appendix S1. Supporting Information [file CNR2-4-e1383-s001.zip › CNR2_1383_SUPPORTING_INFORMATION_supplementary datafile 2.docx]

SUPPORTING INFORMATION

Diet information (supplementary data/file 2)

- Patient 1: The KD LQ formula was stepwise introduced to a maximum of seven exchanges of 100 mL Ketocal LQ 4:1 ratio and 200 kcal. On day 3, he showed hypoglycemia (glucose level, 2.4 mmol/L) combined with hyperketosis (ketone level, 7.2 mmol/L). Because he vomited and refused the LQ formula, the diet was stepwise changed into a KD of 1.6:1 diet ratio with solid food consisting of 48 grams of carbohydrates and MCT (using total of 100 mL MCT fat emulsion/day), which eliminated these symptoms
- Patient 2: The KD LQ formula was stepwise introduced to a maximum of 8.5 exchanges of 100 mL Ketocal LQ 4:1 ratio and 200 kcal and a little snack with an overall diet ratio of 3.8:1. When adequate keto- sis was reached, the diet was stepwise modified into a KD of 2.1:1 diet ratio with solid food consisting of 38 g of carbohydrates and MCT (using total of 110 mL MCT fat emulsion/day).
- Patient 3: She was started on 75% RDA calories 1800 Kcal and 2.5:1 keto ratio. Her calories and keto ratio was fine-tuned at each follow-up to maintain her weight and 4+ ketones She was on low GI and consuming natural glutamine antagonist list food products. She was on MCT oil 20 ml four times a day.
- Patient 4: She was put on ketogenic diet and started on 75% RDA calories 1200 Kcal and 2.5:1 keto ratio. Her calories and keto ratio were fine-tuned at each follow-up to maintain her weight and 4+ ketones.
- Patient 5: The family started under the supervision of a dietician from the United States the KD (modified Atkins diet) at home. The patient was then followed-up by the hospital’s pediatric neurology/epilepsy team. The main strategy of the diet was less than 30-35 g of carbohydrates with solid food and Ketocal LQ 4:1 ratio.
